# Anticancer Dose Adjustment for Patients with Renal and Hepatic Dysfunction: From Scientific Evidence to Clinical Application

**DOI:** 10.3390/scipharm85010008

**Published:** 2017-02-27

**Authors:** Tomi Hendrayana, André Wilmer, Verena Kurth, Ingo GH Schmidt-Wolf, Ulrich Jaehde

**Affiliations:** 1 Institute of Pharmacy, Clinical Pharmacy, University of Bonn, D-53121 Bonn, Germany; a.wilmer@gmx.net (A.W.); v.kurth@uni-bonn.de (V.K.); u.jaehde@uni-bonn.de (U.J.); 2 School of Pharmacy, Institut Teknologi Bandung (ITB), Bandung 40132, Indonesia; 3 Med. Klinik III und Poliklinik, Center for Integrated Oncology (CIO), University of Bonn, D-53127 Bonn, Germany; ingo.schmidt-wolf@ukb.uni-bonn.de

**Keywords:** anticancer, dose adjustment, renal dysfunction, hepatic dysfunction

## Abstract

Most anticancer agents exhibit a narrow therapeutic index, i.e., a small change in plasma concentrations can lead to a less efficacious treatment or an unacceptable degree of toxicity. This study aimed at providing health professionals with a feasible and time-saving tool to adapt the dose of anticancer agents for patients with renal or hepatic dysfunction. A guideline for anticancer agents was developed based on a literature search. An algorithm was generated to enhance the efficiency of the dose adaptation process. Finally, the dosing guideline was converted into an easy-to-use Excel^TM^ tool. The concept was applied to a total of 105 adult patients at the Centre for Integrated Oncology, Bonn, Germany. In total, 392 recommendations for dose adaptation were made and 320 (81.6%) recommendations were responded to by the oncologists. 98.4% of the recommendations were accepted. The algorithm simplifies the decision and screening process for high-risk patients. Moreover, it provides the possibility to quickly decide which laboratory tests are required and whether a dose adjustment for a particular anticancer drug is needed. The Excel^TM^ tool provides a recommended individual dose for patients with renal or hepatic dysfunction. The effectiveness of this strategy to reduce toxicity should be investigated in further studies before being adopted for routine use.

## 1. Introduction

Anticancer agents are characterized by a narrow therapeutic index and large inter-individual pharmacokinetic variability. Therefore a small change in plasma concentration due to organ function impairment may lead to an unacceptable degree of toxicity, interfering with therapeutic outcomes and impeding the success of an anticancer therapy [1,2]. Hence, individually adjusted dosing considering personal conditions and the needs of the patient appears to be crucial for patient safety.

The main determining factors for plasma concentrations of drugs in general and anticancer agents in particular are kidney and liver function, since these organs are responsible for drug metabolism and excretion. Renal and hepatic functions decrease as people become older, leading to the fact that the number of patients with renal or hepatic dysfunction increases with the age. Current studies show that more than 60% of all incidents of cancer and 70% of all cancer-related deaths occur in patients with more than 65 years of age [3]. Keeping in mind that the aging of the population and the process of demographic change in Western countries, the number of elderly cancer patients with insufficient renal or hepatic function will increase within the next decade, bearing a high risk of overdosing and toxicity. Current studies focusing on renal insufficiency in cancer patients show an incidence of 33% for patients with decreased renal function, defined as a glomerular filtration rate (GFR) below 80 mL/min, regardless of age [4].

Clinical practice often copes with these challenges to a very low extent. This may be due to a lack of information about the need for dose adjustment in hepatic or renal dysfunction for a large amount of anticancer compounds. It may also be due to the inconsistency of the literature dealing with this issue, since the approval and marketing of new anticancer drugs frequently proceeds with rather limited information on pharmacokinetics alterations in renal or hepatic impairment [5]. Different sources show high variability in dose recommendations, interfering with adequate dose adjustment [6].

Some researchers, hospitals, and institutions have created guidelines for dose adjustment in organ dysfunction by using a literature search, such as the dosing guidelines for selected anticancer agents in patients with renal or hepatic dysfunction by Lam et al. [7] or the recommendations for dose adjustment for renal and hepatic impairment by the Derby-Burton Cancer Network [8]. Other possibilities for obtaining information about dose adjustment are drug label dosing recommendations or empiric experience.

To overcome the aforementioned problems of adequate dosing in tumor patients with organ dysfunction, a practice-based approach is needed. It has to imply a relief of work for the physician in charge and include evidence-based data and recommendations for specific dose adjustment. Furthermore, the approach has to be compatible with a daily routine.

In this study, we developed a practicable framework for individual dose adjustment for cancer patients undergoing chemotherapy and experiencing renal or hepatic dysfunction for the Centre of Integrated Oncology (CIO) at the University of Bonn. Based on patient observation and a search of the literature, we designed a feasible approach including an anticancer dose adjustment algorithm and a convenient excel tool, facilitating daily work in the hospital. As distinguished from many other studies covering either renal or hepatic dysfunction, our results include data and recommendations for both organ impairments.

Our aim was to provide clinical oncologists and pharmacists with a feasible and timesaving possibility to adapt the dosing of anticancer agents to the individual requirements of patients with renal or hepatic dysfunction in order to maximize the therapeutic outcomes and minimize the toxic effects due to overdosing.

## 2. Materials and Methods

### 2.1. Literature Search

A literature search was conducted, including clinical trials and reviews investigating the influence of organ dysfunction on the pharmacokinetics and toxicity of anticancer agents and including available reviews and guidelines [7,8,9]. Moreover, summaries of product characteristics (SPCs) or drug monographs, e.g., DrugDex^TM^ or the Cancer Centre Ontario Formulary [10], were checked for information regarding dose adaptation in organ dysfunction.

### 2.2. Algorithm Development

Based on practical experience, an algorithm for dose adaptation in organ dysfunction was developed. The purpose was to enhance the efficiency of the dose adaptation process. Anticancer agents were grouped according to lab data necessary for dose calculation. Furthermore, thresholds for the crucial lab data were identified by reviewing the literature and were integrated into the decision tree. Thus, the rapid identification of the necessary lab data for a specific drug combined with “trigger values” for the queried lab data allowed the rapid identification of high-risk patients in need of dose adjustment of a specific anticancer agent.

### 2.3. Excel Tool

In a final step, an easy-to-use Excel tool was generated from the dose adaptation guideline by using the software Microsoft Office Excel^TM^ 2007 (Microsoft, Redmond, WA, USA). An entry mask was created for every anticancer agent included in the guidelines, inquiring into all necessary data for dose calculation. The necessary steps for dose calculation were programmed in the Excel sheet using macros. Renal function is calculated automatically based on the entered demographic and lab data using the equation of Cockcroft and Gault (see guideline application). The calculated or measured values associated with organ function are matched to the guideline for the queried anticancer agent in a further step, and a dose recommendation is provided. A validation of the final tool was done by independent pharmacists.

### 2.4. Guideline Application

Guideline application was conducted on the medical ward for outpatients in the CIO, University of Bonn, Germany, and included all patients with a prescription of chemotherapy at the CIO from May until December 2009. Demographic data (weight, height, sex, age, diagnosis, therapy regiment), current laboratory data (serum creatinine (SCr), total bilirubin (T.Bil.), aspartate aminotransferase (AST), alanine aminotransferase (ALT)), and prescribed anticancer agents (name, standard dose, given dose, reasons for dose reduction) were collected from the medical file of each patient by a clinical pharmacist one day before the next therapy cycle using a standard data collection form. Estimations of renal function were made by a calculation of creatinine clearance (CrCl) from SCr using the equation of Cockcroft and Gault [11]:

(1) $$CrCl\;\left(\frac{ml}{min}\right)=\frac{k\times \left[\left(140-age\right)\times weight\;\left(kg\right)\right]}{SCr\;\left(\frac{mg}{dl}\right)\times 72}$$

where *k* = 1 (male) or 0.85 (female).

Based on the calculated CrCl, the demographic, laboratory, and therapy information and according to the dose adaptation guideline, an individual dose recommendation for each patient was provided by the pharmacist to the physician in charge. This was carried out in written form. The physician had to decide whether to accept or refuse the dose recommendation and had to respond to it in written form. In the case of a rejection, the physician was asked to give the reason for his decision.

## 3. Results

### 3.1. Development of the Dose Adaption Guideline

Depending on CrCl or lab data for liver function like T.Bil., AST, or ALT, specific dose recommendations were provided, including full dose recommendations, recommendations in per cent, specific recommendations in milligram per square meter, and recommendations to omit the dose. For 45 selected anticancer agents, the dose adjustment guidelines for renal and/or hepatic dysfunction are shown in Table 1.

### 3.2. Algorithm Development

**Figure 1:**
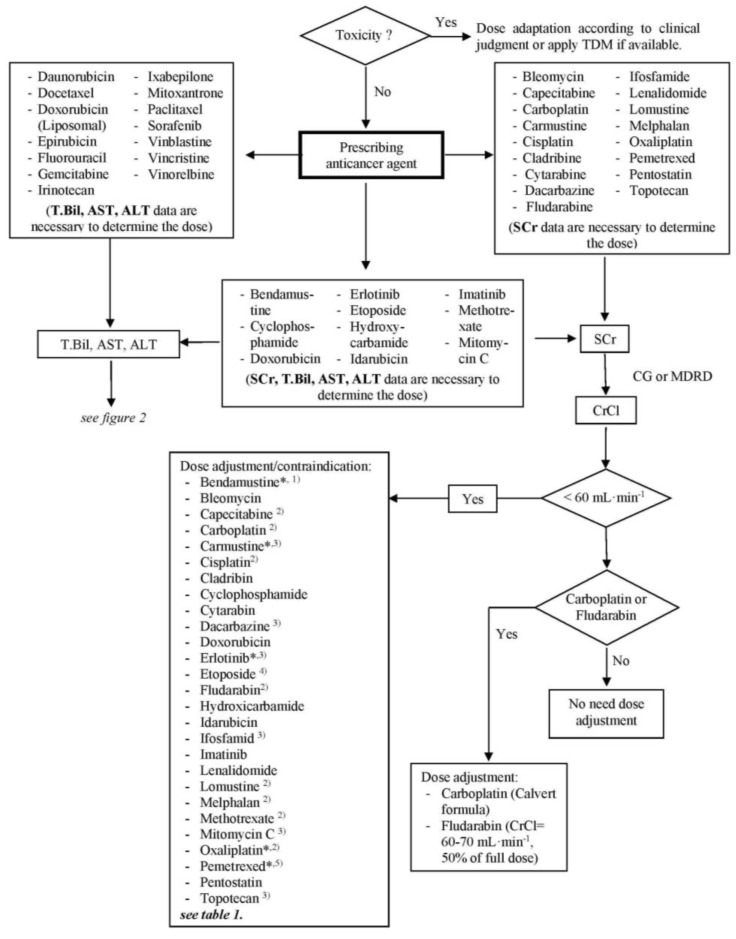
Algorithm of dose adjustment of anticancer agents for a patient with renal dysfunction. TDM, therapeutic drug monitoring; SCr, serum creatinine; T.Bil, total bilirubin; AST, aspartate aminotransferase; ALT, alanine aminotransferase; CrCl, creatinine clearance; CG, Cockcroft & Gault formula; MDRD, simplified Modification of Diet in Renal Disease formula; * use full dose if not contraindicated; ^(1)^ contraindicated if CrCl < 40 mL∙min^−1^; ^(2)^ contraindicated if CrCl < 30 mL∙min^−1^; ^(3)^ contraindicated if CrCl < 10 mL∙min^−1^; ^(4)^ contraindicated if CrCl < 15 mL∙min^−1^; ^(5)^ contraindicated if CrCl < 45 mL∙min^−1^.

**Figure 2:**
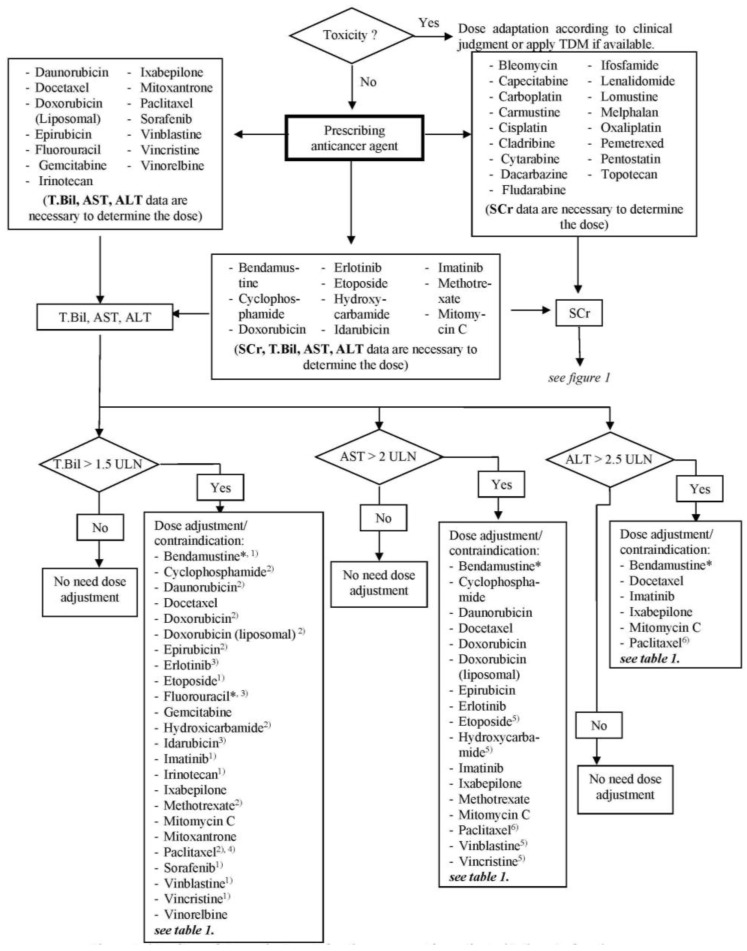
Algorithm of dose adjustment of anticancer agents for a patient with hepatic dysfunction. TDM, therapeutic drug monitoring; SCr, serum creatinine; T.Bil, total bilirubin; AST, aspartate aminotransferase; ALT, alanine aminotransferase; ULN, upper limit normal; * use full dose if not contraindicated; ^(1)^ contraindicated if T.Bil > 3 ULN; ^(2)^ contraindicated if T.Bil > 5 ULN; ^(3)^ contraindicated if T.Bil > 7 ULN; ^(4)^ for Paclitaxel, 24-h infusion and first course of therapy, contraindicated if T.Bil > 7.5 ULN; ^(5)^ contraindicated if AST > 6 ULN; ^(6)^ contraindicated if transaminase > 10 ULN.

The final algorithm for renal dysfunction is shown in Figure 1 and for hepatic dysfunction in Figure 2. For each anticancer drug, laboratory tests, which are crucial for dose adjustment decisions, are stated. For renal function, a threshold of 60 mL/min for CrCl was generated in accordance with the reviewed literature as a point of reference for the discrimination of renally impaired patients. The generated threshold for hepatic function was defined as T.Bil. > 1.5 × ULN (upper limit of normal), AST > 2 × ULN and/or ALT > 2.5 × ULN based on the threshold values for the anticancer agents used in the CIO and the dose adaptation guideline. Patients with renal clearance lower and/or lab data higher than the above specified values are defined as among the high-risk population and should get close monitoring or a dose adaptation. The algorithm provides the possibility to decide quickly which lab parameter is necessary for a decision about dose adaptation and whether or to what extent a dose adjustment for a specific anticancer agent is necessary or not.

### 3.3. Excel Tool

**Figure 3:**
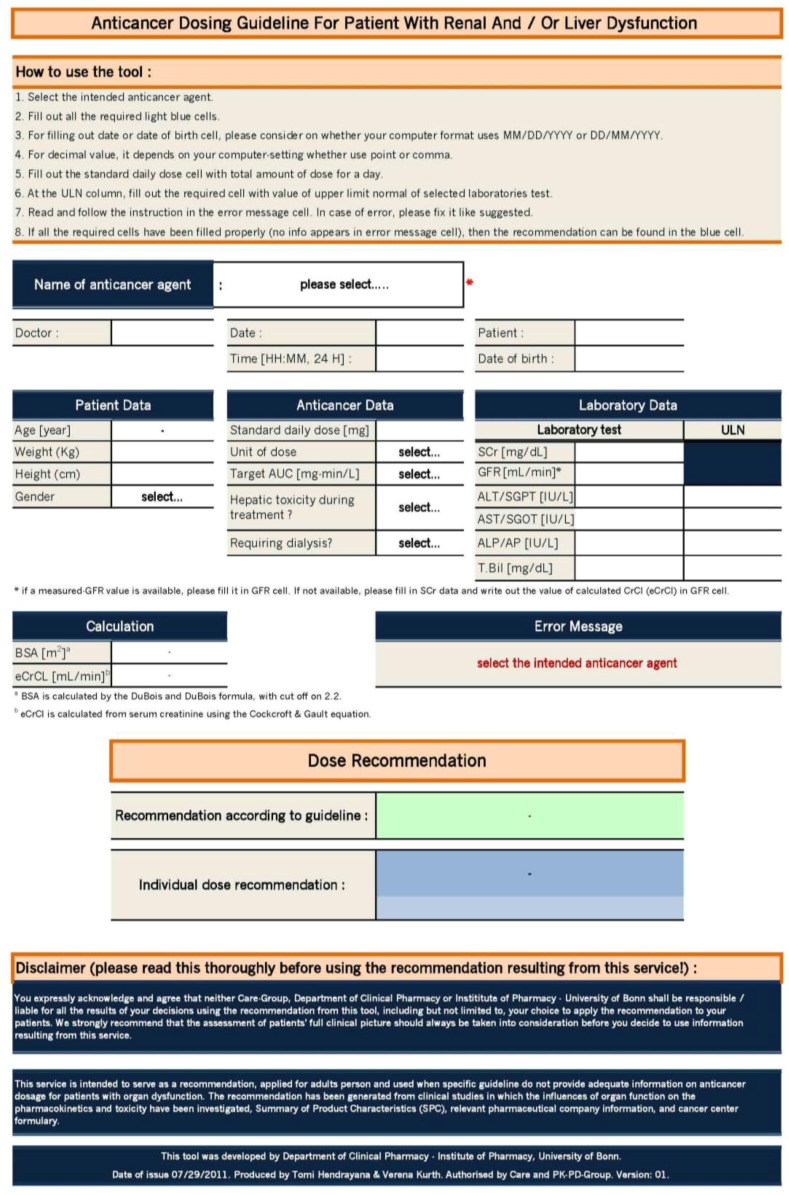
Easy-to-use Excel tool.

For practical-use purposes, an easy-to-use Excel tool was developed in a final step based on the dose adaptation guidelines, as shown in Figure 3. Depending on the queried anticancer agent, the user is asked to insert all data crucial for a dose adjustment calculation for the particular drug. Inquired data may be specific patient characteristics (e.g., age, weight, height and gender), laboratory data (e.g., SCr, ALT, AST, ALP, T.Bil., GFR), and anticancer therapy information (e.g., standard daily dose, unit of dose, target AUC (area under the curve), if hepatic toxicity occurred during treatment, if dialysis is required). Based on the entered data, a precise individual dose recommendation is provided instantly in milligrams per day. Specific error messages give feedback if not all the required cells are filled in correctly or some information is missing.

The tool was validated by three independent clinical pharmacists from the Institute of Pharmacy, University of Bonn. A total of 46 fictitious cases were used for validation; that is at least one case covering each anticancer agent. The validation was successful, given that the calculated results from the Excel tool conformed to the guideline recommendations for each case. No problems emerged, and all reviewers rated the tool as feasible and easy to use.

### 3.4. Guideline Application and Acceptance

The dose adaptation guideline was applied to a total of 105 adult patients. Among the 105 patients, 64.8% were male. The median age was 66 years (mean 61.5; range 22–90 years), and 54.3% were elderly patients (≥65 years). Complete patient characteristics are shown in Table 2.

For each prescription, a recommendation was given based on the dose adaptation guideline, if all necessary demographic and lab data (SCr, T.Bil., AST, body weight, and height) were available. A total of 392 recommendations were made and 320 recommendations (81.6%) were responded to by the physicians. Of the recommendations responded to, a proportion of 98.4% was accepted by the physicians. Only five recommendations were rejected, and the reason for rejection was that the CrCl had only slightly decreased below the guideline limit. In this case, the physician, based on his experience, was of the opinion that the patient could tolerate the full dose.

## 4. Discussion

To our knowledge, this is the first time an algorithm that has the potential to facilitate the dose adaptation process in clinical routine has been developed. Unlike other approaches, it covers both renal and hepatic impairment and permits individual dose adaptation recommendations for cancer patients with organ dysfunction in a feasible and timesaving manner. Potential toxicity due to accumulation effects can be prevented in these patients, and, therefore, the tolerability and safety of anticancer treatment can be improved. The development of the easy-to-use Excel tool takes account of the advancing process of implementing electronic devices and software into daily medical care in hospitals. It is applicable for integration into prescription and dispensing software addressing oncologists and clinical pharmacists. The easy-to-handle entry mask and the software-based calculation save time and prevent entry and calculation errors potentially occurring during the demanding every day care process.

Nevertheless, like other tools, our tool has some limitations. The calculation and determination of renal and hepatic function is based on clinical lab data. For our algorithm, we relied on the SCr concentration for the calculation of renal function by CrCl using the equation of Cockcroft and Gault. Like other studies, we found that relying only on SCr concentration without using prediction equations to determine the patients with renal impairment would cause a great underestimation of the number of affected patients [52]. Calculating CrCl with prediction equations is not at all an exact measure of the GFR but only an estimate. SCr concentrations show a certain variability among individuals due to a great dependency on dietary intake, total muscle mass, medication interfering with renal creatinine handling, age, obesity, cachexia, and diseases [53,54]. Given that an exact measurement of GFR using inulin or isotopic substances may be costly and complex and in general is not used in clinical practice and since there are several studies that validated the prediction equations in different patient populations, our approach to estimating CrCl via the Cockcroft-Gault equation is in accordance with daily clinical practice [53,55,56]. Defining a CrCl of 60 mL/min as the threshold for renal dysfunction is in accordance with the National Cancer Institute (NCI) organ dysfunction protocol templates, and dose modification is crucial below this value for safety and efficacy reasons [57].

To determine liver function, we considered the clinical parameters total bilirubin, AST, and ALT. Measuring these parameters is a typical way of assessing liver function in studies and clinical practice, allowing an estimation of liver condition, integrity, and general metabolic function. Especially elevated serum bilirubin concentrations can specifically indicate liver damage and functional loss [58]. What is not possible by an assessment of these parameters is the prediction of the metabolising enzyme capacity of specific drugs [54]. To overcome this problem, enzyme expression or activity tests could be taken into consideration, which have been developed for several enzymes [59]. In addition to the aforementioned clinical parameters, there are other criteria for the description of liver function based on lab data and symptoms. Among these are the Child-Pugh score and the National Cancer Institute Organ Dysfunction Working Group (NCI-ODWG) criteria. To assure consistent dose adaptation recommendations in hepatic impairment, standard criteria for liver function measurement should be defined [60,61].

The recommendations for dose adaptation emerging from our algorithm are based solely on the aforementioned clinical parameters. They are appropriate only to determinate renal and hepatic function. Every other condition and circumstance justifying dose adaptation, except for organ dysfunction, is not covered by this algorithm. This applies particularly to the occurrence of toxicity during chemotherapy. In this case, the dose has to be reduced with respect to patient safety, regardless of the recommendations given by the dose adaptation guideline. Being aware of this issue, we considered it in the first decision point of the algorithm, which asks for the occurrence of toxicity.

To avoid toxicity and assure the therapeutic effect of anticancer agents, therapeutic drug monitoring (TDM) is probably the best alternative [54,62]. This could also be combined with pharmacokinetic-pharmacodynamic (PK/PD) modelling for a dosing guideline based on toxicity predictions [59]. However, due to the complexity of TDM and PK/PD models, in case of toxicity, the dose typically is decreased according to the physician’s discretion in clinical practice. Further research projects should focus on dose adaptation in cancer patients in the case of occurrence of toxicity.

The risk of under-dosing due to toxicity-associated dose reduction cannot be prevented by our guideline entirely, but, by preventing overdosing due to organ impairment at the beginning of chemotherapy, the risk of toxicity will be decreased. Hence, decreasing the risk of toxicity will also decrease the risk of under-dosing due to toxicity-associated dose reduction. Therefore, under-dosing could be prevented by adapting the dose to the organ function at the beginning of chemotherapy. In case of a toxicity occurrence during therapy, the physician has to consider a dose reduction, regardless of the algorithm.

Our research pointed out an approach to transfer scientific evidence to clinical routine and enhance safety in anticancer drug therapy. The question of whether toxicity can be prevented, or at least reduced, by adopting the tool in clinical practice can only be answered in a randomized controlled trial (RCT) including an adequate number of cancer patients with organ dysfunction. The guideline was pretested and applied in a sample of 105 patients in one hospital, but the sample size was too small to generate significant results of efficacy. The developed algorithm and the Excel tool have not been yet assessed in practice. Their feasibility and usability have to be demonstrated by routinely implementing them in a clinical practice setting on oncologic wards by physicians and clinical pharmacists.

## 5. Conclusions

A dose adaptation guideline for anticancer agents in patients with organ dysfunction was developed, followed by a dosing algorithm and an easy-to-use Excel tool. The algorithm simplifies the decision and screening process for high-risk patients. Moreover, it provides the possibility of quickly deciding which lab parameters are required and whether a dose adjustment for a particular anticancer agent is needed. The Excel tool provides a recommended individual dose. The effectiveness of our method to reduce toxicity should be assessed in a randomized controlled trial.

## Figures and Tables

**Table 1:** Dosing guidelines of selected anticancer agents for patients with renal and/or hepatic dysfunction.

No.	Agent	Dose Adjustment in		Ref.
		Renal Dysfunction	Hepatic Dysfunction	
1.	Bendamustine	Renal impairment: CrCl < 40 mL/min, omit	Moderate (transaminase 2.5–10 × ULN **and** T.Bil 1.5–3 × ULN) or severe (T.Bil > 3 × ULN), omit	[12]
2.	Bleomycin	CrCl 40–50 mL/min, 70% of full dose CrCl 30–40 mL/min, 60% of full dose CrCl 20–30 mL/min, 55% of full dose CrCl 10–20 mL/min, 45% of full dose CrCl 5–10 mL/min, 40% of full dose	No adjustment is required	[9,13,14]
3.	Capecitabine	CrCl 30–50 mL/min, 75 % of full dose CrCl < 30 mL/min, omit	No adjustment is required	[7,9,15,16]
4.	Carboplatin	Dose based on GFR, using Calvert formula: Dose (mg) = target AUC × (GFR + 25). AUC = 5–7 For ESRD patient, CrCl < 30 mL/min, omit	No adjustment is required	[8,17,18,19]
5.	Carmustine	CrCl 45–60 mL/min, 80% of full dose CrCl 30–45 mL/min, 75% of full dose CrCl < 30 mL/min, 70% of full dose	Dosage adjustment maybe necessary; no specific recommendations found	[20,21]
6.	Cisplatin	CrCl 46–60 mL/min, 50 % of full dose CrCl 31–45 mL/min, 25 % of full dose CrCl ≤ 30 mL/min, omit	No adjustment is required	[7,8,22]
7.	Cladribine	CrCl 10–50 mL/min, 75% of full dose CrCl < 10 mL/min, 50% of full dose	No specific recommendations found	[8,23]
8.	Cyclophosphamide	CrCl < 10 mL/min, 50 % of full dose	T.Bil > 3.1–5.0 mg/dL or AST > 180 IU/L, 75% of full dose T.Bil > 5.0 mg/dL, omit	[7]
9.	Cytarabine	CrCl 40–60 mL/min: - if dose > 2 g/m^2^/dose, ↓ to 1 g/m^2^/dose - if dose = 0.75–1 g/m^2^/dose, ↓ to 0.5 g/m^2^/dose CrCl < 40 mL/min: - if dose > 0.75 gm/m^2^/dose, give≤ 200 mg/m^2^/day	No specific recommendations found. Patient with liver dysfunction receiving cytarabine should be carefully monitored and adjust the dose based on clinical judgment.	[7]
10.	Dacarbazine	CrCl 30–60 mL/min, 75% of full dose CrCl 10–30 mL/min, 50% of full dose CrCl < 10 mL/min, omit	No specific recommendations found. Patient with liver dysfunction receiving dacarbazine should be carefully monitored and adjust the dose based on clinical judgment.	[7]
11.	Daunorubicin	Serum creatinine > 3 mg/dL, 50% of full dose	T.Bil 1.5–3.0 mg/dL or AST 60–180 IU/L, 75% of full dose T.Bil > 3.1–5.0 mg/dL or AST > 180 IU/L, 50% of full dose T.Bil > 5.0 mg/dL, omit	[7]
12.	Docetaxel	No adjustment is required	Transaminase >1.5 × ULN, **and** ALP > 2.5 ULN, the recommended dose is 75mg/m^2^ T.Bil > ULN **and/or** transaminase > 3.5 × ULN associated with ALP > 6 × ULN, should not be used unless strictly indicated	[8,9,24,25]
13.	Doxorubicin	CrCl < 10 mL/min, 75% of full dose	T.Bil 1.5–3.0 mg/dL or AST 60–180 IU/L, 50% of full dose T.Bil > 3.1–5.0 mg/dL or AST > 180 IU/L, 25% of full dose T.Bil > 5.0 mg/dL, omit	[7,9]
14.	Doxorubicin (Liposomal)	No adjustment is required	T.Bil 1.2–3.0 mg/dL or AST 60–180 IU/L, 50% of full dose T.Bil > 3.0 mg/dL or AST > 180 IU/L, 25% of full dose T.Bil > 5.0 mg/dL, omit	[7,26]
15.	Epirubicin	Serum creatinine > 5 mg/dL, lower doses should be considered	T.Bil 1.2–3.0 mg/dL or AST 2–4 × ULN, 50% of full dose T.Bil > 3.0 mg/dL or AST > 4 × ULN, 25% of full dose T.Bil > 5.0 mg/dL, omit	[7,9]
16.	Erlotinib	CrCl < 10 mL/min, omit	AST ≥ 3 × ULN or T.Bil 1–7 mg/dL, 50% of full dose. T.Bil > 7.0 mg/dL, omit	[27,28]
17.	Etoposide	CrCl > 15–50 mL/min, 75% of full dose CrCl < 15 mL/min, omit	T.Bil 1.5–3 mg/dL or AST 60–180 units, 50% of of full dose T.Bil ≥ 3 mg/dL or AST > 180 units, omit	[7,8,9,29]
18.	Fludarabine	CrCl 30–70 mL/min, 50% of full dose CrCl < 30 mL/min, omit	No adjustment is required	[8,30]
19.	Fluorouracil	No adjustment is required	T.Bil > 5.0 mg/dL, omit	[7,31]
20.	Gemcitabine	No specific recommendations found	Increased AST: no need dose adjustment Increased T.Bil: reduce dose by 20% (i.e., from 1000 to 800 mg/m^2^) and increase if tolerated.	[9,27,32]
21.	Hydroxyurea/Hydroxycarbamide	CrCl 10–60 mL/min, 75% of full dose CrCl < 10 mL/min, 50% of full dose	T.Bil 1.5–5.0 mg/dL or AST 60–180 IU/L, 50% of full dose T.Bil > 5.0 mg/dL or AST > 180 IU/L, omit	[7]
22.	Idarubicin	Serum creatinine ≥ 2.5 mg/dL, dose reduction recommended.	T.Bil 2.5–5.0 mg/dL, 50% of full dose T.Bil > 5.0 mg/dL, omi.	[7,33]
23.	Ifosfamide	CrCl 46–60 mL/min, 80% of full dose CrCl 31–45 mL/min, 75% of full dose CrCl 10–30 mL/min, 70% of full dose CrCl < 10 mL/min, omit	No specific recommendations found	[7,9]
24.	Imatinib	CrCl of 40 to 59 mL/min, doses > 600 mg are not recommended CrCl 20 to 39 mL/min, 50% of full dose. Dose can be increased up to max. 400 mg CrCl < 20 mL/min, use with caution (2 patients with severe renal impairment, doses of 100 mg/day were tolerated)	Initial dose: - severe hepatic impairment, initial dose: 75% of full dose. Hepatic toxicity during treatment: - transaminases > 5 × ULN or T.Bil > 3 × ULN; omit. Restart at reduced doses (reduce from 400 mg to 300 mg, from 600 mg to 400 mg, or from 800 mg to 600 mg) when transaminases < 2.5 × ULN **and** T.Bil < 1.5 × ULN.	[34]
25.	Irinotecan (Weekly, usual dose 125 mg/m^2^ for 4 of 6 weeks)	No adjustment anticipated to be required	Increased AST: no need dose adjustment. T.Bil 1.5–3 × ULN **and** ratio of AST to ALT < 5 × ULN, 60 mg/m^2^ T.Bil 3.1–5 × ULN **and** ratio of AST to ALT < 5 × ULN, 50 mg/m^2^ T.Bil < 1.5 × ULN **and** ratio of AST to ALT 5.1–20 × ULN, 60 mg/m^2^ T.Bil 1.5–3 × ULN **and** ratio of AST to ALT 5.1–20 × ULN, 40 mg/m^2^.	[27,35]
26.	Irinotecan (3 weekly, usual dose 350 mg/m^2^ every 3 weeks)	No adjustment anticipated to be required	Increased AST: no need dose adjustment T.Bil > 1.5–3 × ULN, dose = 200 mg/m^2^ T.Bil > 3 × ULN, omit	[27,35,36]
27.	Ixabepilone (monotherapy)	No specific recommendations found	T.Bil < 1.5 × ULN **and** AST < 10 ULN **and** ratio AST to ALT < 10 × ULN), reduce the dose to 32 mg/m^2^ T.Bil 1.5–3 × ULN **and** transaminase < 10 × ULN, dose 20 mg/m^2^; may escalate dose up to 30 mg/m^2^ maximum in subsequent cycles, if tolerated	[37]
28.	Ixabepilone (in combination with capecitabine)	No specific recommendations found	T.Bil > ULN or transaminase > 2.5 × ULN, omit	[37]
29.	Lenalidomide (use for myelodysplastic syndrome/MDS)	CrCl 30–60 mL/min, 5 mg every 24 h CrCl < 30 mL/min (not requiring dialysis), 5 mg every 48 h CrCl < 30 mL/min (requiring dialysis), 5 mg 3 times a week after each dialysis	No specific recommendations found	[38,39]
30.	Lenalidomide (use for Multiple Myeloma/MM)	CrCl 30–60 mL/min, 10 mg every 24 h CrCl < 30 mL/min (not requiring dialysis), 15 mg every 48 h CrCl < 30 mL/min (requiring dialysis), 5 mg once daily, dose after dialysis on dialysis days	No specific recommendations found	[38,39]
31.	Lomustine	CrCl 45–60 mL/min, 75% of full dose CrCl 30–45 mL/min, 70% of full dose CrCl < 30 mL/min, omit	No specific recommendations found	[8,40]
32.	Melphalan	CrCl 45–60 mL/min, 85% of full dose CrCl 30–45 mL/min, 75% of full dose CrCl 10–30 mL/min, 70% of full dose CrCl < 10 mL/min, 50% of full dose	No adjustment is required	[7,40,41]
33.	Methotrexate	For low dose (<1 g/m^2^): CrCl 30–60 mL/min, 50 % of full dose CrCl < 30 mL/min, omit For high dose (> 1 g/m^2^) used, consider to conduct Therapeutic Dose Monitoring (TDM)	T.Bil 3.1–5.0 mg/dL or AST > 180 IU, 75% of full dose T.Bil > 5.0 mg/dL, omit	[7,8]
34.	Mitomycin C	CrCl 30–60 mL/min, 75% of full dose CrCl 10–30 mL/min, 50% of full dose CrCl < 10 mL/min, omit	T.Bil 1.5–3.0 mg/dL, 50% of full dose T.Bil > 3.0 mg/dL or transaminase > 3 × ULN, 25% of full dose	[7]
35.	Mitoxantrone	No adjustment is required	T.Bil 1.5-3.0 mg/dL, 50% of full dose T.Bil > 3.0 mg/dL, 25% of full dose	[7,42]
36.	Oxaliplatin	CrCl < 30 mL/min, omit	No adjustment is required	[9,15,27,43]
37.	Paclitaxel (3-h infusion and first course of therapy)	No adjustment is required	Transaminase < 10 × ULN **and** T.Bil 1.26–2 × ULN, dose = 135 mg/m^2^ Transaminase < 10 × ULN **and** T.Bil 2.01–5 × ULN, dose = 90 mg/m^2^ Transaminase ≥ 10 × ULN or T.Bil > 5 × ULN, omit	[44,45]
38.	Paclitaxel (24-h infusion and first course of therapy)	No adjustment is required	Transaminase of 2–10 × ULN **and** T.Bil < 1.5 mg/dL, dose = 100 mg/m^2^ Transaminase < 10 × ULN **and** T.Bil 1.6–7.5 mg/dL, dose = 50 mg/m^2^ Transaminase ≥ 10 × ULN or T.Bil > 7.5 mg/dL, omit	[43,44]
39.	Pemetrexed	CrCl < 45 mL/min, omit	No specific recommendations found	[21,46]
40.	Pentostatin	CrCl 45–60 mL/min, 70% of full dose CrCl 30–45 mL/min, 60% of full dose CrCl < 30 mL/min, consider to use alternative drugs if possible	Not applicable	[7]
41.	Sorafenib	No adjustment is required	T.Bil ≤ 1.5 × ULN: 400 mg twice a day T.Bil 1.5–3 × ULN: 200 mg twice a day T.Bil > 3 × ULN: omit	[27,47]
42.	Topotecan	CrCl 30–60 mL/min, 75% of full dose CrCl 10–30 mL/min, 50 % of full dose CrCl < 10 mL/min, omit	No adjustment is required	[7,9]
43.	Vinblastine	No adjustment is required	T.Bil 1.5–3.0 mg/dL or AST 60–180 IU/L, 50% of full dose T.Bil > 3.1 mg/dL or AST > 180 IU/L, omit	[7,48]
44.	Vincristine	No adjustment is required	T.Bil 1.5–3.0 mg/dL or AST 60–180 IU/L, 50% of full dose T.Bil > 3.1 mg/dL or AST > 180 IU/L, omit	[7,49]
45.	Vinorelbine	No adjustment is required	T.Bil 2.1–3 × ULN: 50% of dose T.Bil > 3 × ULN: 25% of dose	[7,27,50,51]

Ref., references; CrCl, creatinine clearance; AST, aspartate aminotransferase; ALT, alanine aminotransferase; ULN, upper limit normal; T.Bil, total bilirubin; GFR, glomerular filtration rate; AUC, area under the curve; ESRD, end stage renal disease; ALP, alkaline phosphatase.

**Table 2:** Patient characteristics (N = 105).

Characteristics	No. of Patients	Percentage
Sex		
Female	37	35.2%
Male	68	64.8%
Age, years		
Mean Age	61.5	
Median Age	66	
Range	22–90	
Group of age		
Adult	48	45.7%
Elderly (≥65 years)	57	54.3%
Site of cancer prevalence		
Billiary tract	7	6.7%
Breast	4	3.8%
Colorectal	23	21.9%
Esophageal	6	5.7%
Gastric	10	9.5%
Lymphoma	16	15.2%
Myeloma	4	3.8%
Pancreatic	13	12.4%
Testicular	6	5.7%
Urethra	6	5.7%
Others *	10	9.5%

* bladder cancer, leukemia, lung cancer, prostate cancer and ITP (idiophatic thrombocytopenic purpura).
